# Coaching beyond the game: a qualitative study of volunteer coaches in European grassroots esports

**DOI:** 10.3389/fspor.2026.1913015

**Published:** 2026-08-28

**Authors:** André Baumann, Loizos Georgiou, Robert Juul Jønsson, Matthew Watson, Ståle Pallesen, Rune Aune Mentzoni

**Affiliations:** 1 The Norwegian Esports Federation, Oslo, Norway; 2 Bredde-e-sport Alliansen, Oslo, Norway; 3 Danske Gymnastik- og Idrætsforeninger, Copenhagen, Denmark; 4 Department of Performance Psychology, German Sport University Cologne, Cologne, Germany; 5 Department of Psychosocial Science, University of Bergen, Bergen, Norway

**Keywords:** coach education, eSports, esports coaches, grassroots esports, inclusion, qualitative research, volunteer coaches, youth development

## Abstract

**Introduction:**

Grassroots esports is increasingly organised through sports clubs and community-based initiatives for children and young people, yet little is known about how volunteer coaches understand and describe their roles in these settings. Existing esports coaching research has primarily focused on performance-oriented contexts, leaving limited insight into how coaches make sense of grassroots settings where participation, inclusion, social development, and safe digital cultures may be as important as technical skill development. This qualitative study explored how volunteer grassroots esports coaches in Norway, Denmark, and the Netherlands understood and described their coaching roles.

**Methods:**

Seventeen coaches participated in semi-structured online interviews conducted between March and May 2026. Data were analysed using reflexive thematic analysis.

**Results:**

The analysis generated three themes. First, coaches primarily understood their role as youth-oriented social facilitators rather than game-specific technical instructors, emphasising adult presence, safe participation, positive behaviour, and inclusive environments. Second, coaches described how limited equipment, volunteer availability, access to formal coach education, and practical coaching resources constrained their ability to provide structured and meaningful activities. Third, health and lifestyle guidance occupied a marginal and weakly systematised position in everyday coaching practice. While energy drinks were commonly managed through clear rules, broader topics such as sleep, nutrition, physical activity, and recovery were often perceived as outside the scope of the coaching role or difficult to address with players.

**Discussion:**

Overall, the findings suggest that grassroots esports coaching is best understood as a hybrid practice situated between sport coaching, youth work, digital culture, and community volunteering. Strengthening grassroots esports coaching may benefit from accessible coach education, practical session resources, peer-learning arenas, and clear guidance on inclusion, online communication, player behaviour, and health-related boundaries, rather than simply transferring elite esports coaching models to grassroots settings.

## 1 Introduction

Esports is commonly understood as organised competitive digital gaming in which players' actions and outcomes are mediated through electronic systems and human–computer interfaces, although definitions vary (1, 2). Over time, esports has developed from relatively informal competitive gaming cultures into increasingly organised forms of digital competition, shaped by both grassroots initiatives and commercial actors (3, 4). In this study, grassroots esports refers to organised, non-professional esports activities delivered through club-based or community-oriented structures for children and young people. The term distinguishes grassroots esports from elite, academy, and commercial high-performance esports, while recognising that grassroots activities may still involve structured training, competition, and game-specific skill development. This distinction is important because grassroots esports is not merely a lower competitive tier, but a setting shaped by different organisational purposes.

Grassroots esports should therefore be understood not only as a stepping stone into competitive play, but also as a local youth and community-based practice. Indeed, research on regional esports markets demonstrates how local actors can support offline social spaces that enable young participants to meet in person and build social connections around gaming (5). Studies of grassroots esports activism similarly show that local actors often seek to legitimise esports by framing it as a socially responsible and developmentally meaningful activity for young people (6). These perspectives align with recent research on Norwegian grassroots esports clubs, where esports was framed as a safe social arena for children and young people, but also as an activity constrained by limited organisational and pedagogical capacity (7). At the same time, sustainability research suggests that the sector still struggles with foundational challenges, particularly regarding health, inclusion, and formal governance (8). Taken together, this literature suggests that the successful implementation of grassroots esports depends not only on games, equipment, and tournaments, but also on adults and local organisations that translate competitive gaming into safe, inclusive, and developmentally meaningful practice.

Although adults, coaches, and local organisations play a key role in making grassroots esports safe and meaningful for young people, esports coaching research has primarily examined coaching in performance-oriented contexts. For the purposes of this study, grassroots esports coaching refers to the facilitation of organised esports activities for children and young people in voluntary, club- or community-based settings. The coach role is understood broadly, encompassing not only game-specific instruction, but also the structuring of sessions, support for player learning, management of communication and behaviour, and the creation of safe and inclusive social environments.

Existing esports coaching literature suggests that effective coaching draws on more than game expertise alone. Factors such as pedagogical knowledge and interpersonal skills have been identified as important; for example, coaches may draw on verbal communication, game review practices, and constraints-led approaches (9–11). Esports coaching has also been described as involving communication, leadership, decision-making support, performance analysis, and player-development strategies, while taking place within complex digital and technologically mediated environments. Existing research also highlights how coach learning is often informal, the role is only weakly formalised through training and qualification systems, and traditional forms of coach education are generally devalued among stakeholders (12, 13). This suggests that esports coaches may be expected to support structured learning and player development in a field where coach education pathways, role expectations, and professional standards remain comparatively uncodified. What remains less clear is how coaching is understood and described in grassroots settings, where the coaching role may extend beyond game-specific instruction to encompass broader responsibilities related to safe participation and personal and social development.

Emerging research on scholastic esports similarly suggests that coaches may view esports programmes as spaces for life-skill development, social belonging, confidence, and positive peer environments (14). Youth-development perspectives provide a useful basis for understanding why organised grassroots esports cannot be assumed to be beneficial simply because it is structured or club-based. In youth sport, positive developmental outcomes are understood to depend on the quality of activities, relationships, and settings, rather than participation alone. Positive youth development frameworks commonly emphasise outcomes such as competence, confidence, connection, and character (15), while broader athlete-development perspectives link participation environments to continued participation, performance, and personal development (16). These perspectives support examining grassroots esports coaches not only as technical instructors, but also as adults who help shape the social and developmental conditions under which young people participate in organised gaming.

Traditional sport coaching literature provides a useful lens for understanding this broader role. Côté and Gilbert (17) conceptualise effective coaching as context-dependent and grounded in professional, interpersonal, and intrapersonal knowledge. Importantly, their framework links coaching not only to skill development, but also to athlete outcomes such as confidence, competence, connection, and character. This broader view is also relevant because perceived coaching competence has been associated with athletes' trust in the coach (18). In youth and participation sport, coaches are therefore understood not merely as instructors of technical skills, but as key facilitators who shape whether participation is experienced as supportive, inclusive, and developmentally meaningful (19–21). Athlete-centred coaching perspectives similarly emphasise the importance of supporting participants' autonomy, understanding their individual needs, and creating environments in which they can take an active role in their own development (22). Grassroots esports coaching can be examined through a similar holistic and player-centred lens, because esports participation is not automatically developmental or beneficial. Rather, organised esports activities require intentional facilitation to support positive social environments, manage harmful communication, and address health-related boundaries. This may be particularly important in esports, where players often guide much of their own development outside organised sessions and where participation takes place within digital environments that may require different forms of social, pedagogical, and health-related support than traditional sport. However, because esports coach education remains weakly formalised and grassroots esports coaching is still under-researched, it remains unclear how volunteer coaches understand this broader developmental mandate, which outcomes they prioritise, and whether they draw on a holistic knowledge base in their accounts of coaching.

At the same time, esports coaching cannot be understood simply as sport coaching transferred to a digital setting. It is shaped by digital mediation and game-specific cultures that affect how players practise, communicate, and learn (10, 23, 24). These conditions affect what coaches need to know and do. Game knowledge and prior playing experience may help coaches establish credibility and communicate effectively with players, yet recent research also suggests that such experience can be overvalued when separated from pedagogical skill, interpersonal competence, and the ability to work with diverse groups of players (12). Health-related issues may also become relevant when examining the boundaries of the grassroots esports coaching role. Esports participation has been associated with health-related concerns among some players, particularly around physical strain, sedentary behaviour, insufficient physical activity, sleep, nutrition, and energy drink consumption (25–28). In grassroots settings, however, such concerns should not be assumed to fall straightforwardly within the responsibility of volunteer coaches. Rather, they raise questions about where coaches and clubs draw the boundary between providing safe activity structures and offering more specialised health or lifestyle guidance. Grassroots esports coaching therefore needs to be examined as a hybrid practice situated between sport coaching, youth work, digital culture, and community volunteering.

Against this backdrop, the aim of this study was to explore how volunteer grassroots esports coaches in Norway, Denmark, and the Netherlands understand and describe their coaching roles in organised esports activities for children and young people. In doing so, the study examines how coaches talk about authority and competence, how they describe balancing game-specific instruction with broader youth-oriented responsibilities, and how organisational conditions and peripheral health-related expectations shape their accounts of coaching.

## 2 Method

This study used a qualitative interview design to explore how grassroots esports coaches understand and describe their coaching roles. The study was informed by a contextualist epistemological position, recognising that participants' accounts provide access to their meanings, experiences, and sense-making, while also being shaped by social, cultural, and organisational contexts. In this sense, the analysis did not aim to establish an objective or singular account of grassroots esports coaching, but to develop a theoretically informed interpretation of patterns across the interview material. This position aligns with Madill et al.'s distinction between realist, contextualist, and constructionist approaches to qualitative analysis, and with Braun and Clarke's approach to thematic analysis as a flexible method for identifying, analysing, and reporting patterns of meaning within qualitative data (29, 30). The study should therefore be understood as interpretative rather than evaluative: it examines how coaches make sense of their roles and practices, rather than assessing whether those practices adhere to a predefined model of effective coaching.

The study followed the principles of reflexive thematic analysis, as developed by Braun and Clarke (30) and later elaborated in their quality criteria for reflexive thematic analysis (31). Themes were therefore not treated as pre-existing categories to be discovered in the data, but as analytic constructions developed through repeated engagement with the interview material, coding, comparison, and interpretation. The analysis focused on both semantic content, such as coaches' explicit descriptions of responsibilities, challenges, and practices, and latent patterns concerning role ambiguity, legitimacy, competency gaps, and health-related boundaries. Themes were developed through an iterative interpretative process rather than by code frequency alone, with codes reviewed, clustered into candidate subthemes, and refined into broader themes in relation to the research questions and the overall dataset. Consistent with reflexive thematic analysis, the study did not use coding reliability, interrater agreement, or saturation as quality criteria. Instead, methodological quality was addressed through transparency in reporting, reflexive engagement with the researchers' positions, attention to coherence between data and interpretation, and detailed presentation of participant context, analytic procedures, and illustrative extracts (32–34).

### 2.1 Participants and recruitment

The initial recruitment target was 15 participants. Sample-size planning was guided by the concept of information power, which suggests that the adequacy of a qualitative interview sample depends on the relevance and richness of the information provided in relation to the study aim, sample specificity, use of established theory, quality of dialogue, and analysis strategy (35). Because the study had a focused aim, recruited participants had direct coaching experience in organised grassroots esports, the analysis drew on existing coaching and youth development literature, and semi-structured interviews were suitable for cross-case thematic analysis, a target of approximately 15 participants was considered sufficient for generating a rich and analytically manageable dataset. The study did not use saturation as a formal stopping criterion. Instead, sample adequacy was considered in relation to information power, the specificity of the recruited participants, the richness of the interview accounts, and the ability of the final dataset to support cross-case thematic interpretation of the study aim. After the completion and initial review of the interviews, the final sample was considered analytically sufficient for exploring shared patterns in how volunteer grassroots esports coaches understood and described their roles. No known conflicts of interest or dependent relationships existed between the interviewers and participants, which was considered important for supporting open dialogue during the interviews.

Eligible participants were required to be at least 18 years old, live in Norway, Denmark, or the Netherlands, coach grassroots or local-level esports, and work with children or young people. These criteria were selected to ensure that participants had direct experience of the coaching context under investigation: organised, non-professional esports activities involving children or young people in voluntary, club-based, or community-oriented settings. Participants were recruited through social media channels and email lists associated with DGI, Bredde-e-sport Alliansen, and H20 Esports. DGI also recruited participants through direct contact at a stand during Gamebox 2026. All individuals who accepted an invitation were interviewed. In total, 18 interviews were conducted; however, one Dutch participant was excluded after the interview because they coached other coaches rather than children or young people in grassroots esports. The final sample therefore consisted of 17 unpaid volunteer coaches: nine from Norway, four from Denmark, and four from the Netherlands. The participants coached in local grassroots esports settings that varied in organisational form, including esports initiatives embedded in traditional sports clubs and other community-oriented esports organisations. Additional contextual information about the participants, including age, gender, club context, player age groups, player numbers, and training frequency, is reported in the Results section to support assessment of transferability. The uneven distribution across countries reflects the researchers' stronger access to the Norwegian grassroots esports field, combined with a pragmatic decision to end recruitment in May 2026 to keep the study within its planned March–May 2026 data collection period. As the study was not designed as a comparative cross-national analysis, recruitment aimed to generate information-rich accounts that could illuminate patterns and variations across grassroots esports contexts, rather than aiming for balanced country subsamples. Because participation was voluntary, the sample may overrepresent coaches who were particularly engaged in esports development or motivated to discuss coaching practice.

### 2.2 Data collection procedure

A semi-structured interview guide was developed by the research team to ensure consistency across interviews while allowing participants to elaborate on issues of particular relevance to their coaching context, in line with qualitative guidance on the use of semi-structured interviews to combine focused coverage of key domains with flexibility for participant-led elaboration (36). The guide was informed by the study aim, previous literature on esports coaching, sport coaching, youth development, inclusion, and health-related issues in esports, as well as the researchers' practical familiarity with grassroots esports. In particular, the guide was developed to explore role understanding, sources of competence, session organisation, inclusion, communication, health-related boundaries, and structural conditions, reflecting both existing esports coaching literature and broader youth sport coaching frameworks. It included questions on coach identity, competence, pedagogical practice, inclusion and diversity, health-related boundaries, and structural conditions and support. These domains addressed how coaches understood their role, how they planned and adapted training activities, how they worked with player differences and inclusive participation, and how organisational resources and constraints shaped their coaching. Health-related questions focused more specifically on how coaches understood and approached topics such as physical activity, sleep, nutrition, and energy drink use, as esports participation has been linked to health-related concerns in previous research. The interview guide was used as a flexible structure rather than a purely structured questionnaire, allowing interviewers to tailor follow-up questions to each participant's club context and coaching experience and to probe for examples. Each interview began with a brief explanation of the study aim before moving through the interview guide, with follow-up questions used where relevant. Interviews were conducted digitally via Google Meet or Zoom between March and May 2026 and lasted between 50 and 90 min. Norwegian and Danish interviews were conducted in the participants' national languages, whereas interviews with Dutch participants were conducted in English. All participants received written information about the study and provided informed consent before taking part. With participants' consent, interviews were audio-recorded and transcribed using TurboScribe. Each transcript was subsequently reviewed and corrected by the respective interviewer shortly after the interview to ensure accuracy and preserve contextual meaning. This review was particularly important because the interviews included esports-specific terminology, references to game titles, and context-dependent descriptions of local organisational practices. Audio files were deleted after transcription. The interview guide is available as Supplementary Material.

### 2.3 Researcher reflexivity and methodological integrity

The study was reported with attention to transparency, researcher reflexivity, and methodological integrity in qualitative research (32–34). Interviews were conducted by three members of the research team: AB, RJJ, and LG. The interviewer team had substantial familiarity with esports, both as former or current players and through several years of combined work in grassroots esports. This insider familiarity supported the use of context-sensitive follow-up questions and helped the interviewers interpret esports-specific terminology, game-related references, and organisational practices. At the same time, such familiarity may also have shaped which aspects of participants' accounts were noticed, followed up, and interpreted as analytically meaningful.

The first author conducted the coding and initial theme development. This analytic structure is consistent with reflexive thematic analysis, where themes are understood as interpretative outputs generated through sustained engagement with the data rather than as products of coding consensus. As the first author also has professional expertise in nutrition and health in esports contexts, this background may have sensitised the analysis to health-related issues such as sleep, nutrition, physical activity, and energy drink use. To address this reflexively, health-related findings were interpreted not as evidence that these topics emerged spontaneously as central concerns, but as accounts of how coaches positioned and bounded such issues when explicitly asked about them.

Several steps were used to support methodological integrity. The study aim, recruitment strategy, sample rationale, participant context, interview procedures, transcription process, and analytic approach are described in detail to support transparency and enable readers to assess the transferability of the findings. Transcripts were reviewed and corrected by the respective interviewer shortly after each interview. Candidate themes, selected extracts, and developing interpretations were discussed within the author team during manuscript development. MW additionally contributed as a critical friend, questioning analytic assumptions, considering alternative interpretations, and challenging the boundaries and coherence of candidate themes. These discussions were used to challenge, refine, and clarify the interpretation, rather than to establish interrater reliability or coding consensus. The use of participant quotations and the analytical table was intended to make the link between empirical material, codes, subthemes, and themes visible to readers. Credibility was therefore supported through prolonged engagement with the transcripts, reflexive consideration of researcher positioning, critical analytic dialogue among authors, and transparent reporting of how interpretations were developed. Transferability was supported through contextual description of the participants and grassroots esports settings rather than through claims of statistical representativeness.

### 2.4 Data analysis

Data were analysed using reflexive thematic analysis, guided by Braun and Clarke's six-phase approach: familiarisation with the data, generation of initial codes, searching for candidate themes, reviewing themes, defining and naming themes, and producing the final report (30). Transcripts were imported into MAXQDA 26.2.1, which was used by the first author to organise coding, analytic memos, coded extracts, and candidate theme development (37). In line with later quality criteria for reflexive thematic analysis, the analysis was treated as an interpretative and iterative process rather than a procedure for achieving coding reliability, interrater agreement, or quantifying code frequency (31).

The analysis began with repeated reading of the corrected transcripts to become familiar with the content and context of each interview. Initial codes were then generated across the dataset, focusing on coaches' descriptions of role identity, competence, pedagogical practice, inclusion, organisational conditions, and health-related responsibilities. Coding was both inductive and theoretically sensitised: codes were grounded in participants' accounts, while the first author's attention was also informed by the study aim and by prior literature on coaching, youth development, grassroots esports, and health-related boundaries.

Codes were reviewed, compared, and clustered into candidate subthemes before being refined into broader themes in relation to the research questions and the overall dataset. Theme development involved moving back and forth between coded extracts, individual transcripts, analytic memos, and the full dataset to ensure that the final themes captured coherent and analytically meaningful patterns. During this process, candidate themes were examined in relation to whether they were internally coherent, analytically distinct, and sufficiently supported by extracts across the dataset. Candidate themes were revised where they overlapped too strongly, lacked sufficient analytic focus, or did not adequately capture the relationship between coaching role understandings and the organisational conditions described by participants.

Consistent with a contextualist and reflexive approach, themes were not treated as pre-existing categories to be discovered, but as analytic constructions developed through engagement with the data, the research questions, and relevant literature. The analysis focused primarily on semantic content, while also attending to latent patterns concerning role ambiguity, legitimacy, organisational capacity, competency gaps, and health-related boundaries. Although the sample included coaches from three countries, the study was not designed as a comparative cross-national analysis. Country was nevertheless considered as contextual information during theme development, and coded extracts were reviewed for possible cross-national patterning. No consistent country-specific patterns were identified, and the findings are therefore presented across the full dataset. AI tools were not used for coding, theme development, or interpretation.

### 2.5 Ethics, data, and preregistration

The processing of personal data was assessed and approved by Sikt – Norwegian Agency for Shared Services in Education and Research, reference number 282076. The study included participants from Norway, Denmark, and the Netherlands, and the authors considered applicable national and institutional requirements in the participating countries. No separate local ethics committee approval was obtained in Denmark or the Netherlands. The full interview transcripts are not publicly available due to confidentiality considerations and the potential risk of participant identification within a small and interconnected field. Selected anonymised excerpts are, however, included in the manuscript.

All participants received written information about the study and provided written informed consent before participation. The information letter described the purpose of the study, what participation involved, how data would be processed, and the participants' right to withdraw. Interviews were audio-recorded only with participants' consent. Audio files and transcripts were stored in the University of Bergen's secure research data storage solution, SAFE, with access restricted to authorised members of the research team. Audio files were deleted after transcription. Transcripts were reviewed and corrected by the respective interviewer, and names, club names, locations, and other directly or indirectly identifying details were removed or generalised where necessary. Participant quotations are presented using pseudonyms.

The study was not preregistered. Although preregistration can be adapted for qualitative research, it is not a methodological requirement of reflexive thematic analysis, which involves recursive and interpretative engagement with the data. Methodological integrity was instead supported through transparent reporting and reflexive consideration of how researcher positioning shaped the analytic process.

## 3 Results

### 3.1 Participant context and thematic overview

The participant sample consisted of 17 grassroots esports coaches from different clubs, with an average age of 36 years (range 23–48). The cohort was predominantly male, with one coach identifying as female. Notably, 12 of the 17 coaches were affiliated with esports initiatives organised within traditional sports clubs, placing much of the sample within a voluntary sport-based organisational context. The grassroots clubs catered to a wide demographic, with coaches managing players aged 8–35 years. A common structural approach among the coaches was dividing players into two age tiers: a younger group (averaging 11 years) and an older group (averaging 20 years). Female players were notably underrepresented across the clubs, ranging from zero to eight per club, with an average of two. Most clubs accommodated between 15 and 60 players in total, typically offering two training sessions per week, lasting 90–120 min each. Thematic analysis of the interview data resulted in three overarching themes: 1) The grassroots esports coach as a youth-oriented facilitator; 2) Resource capacity and structure in grassroots esports coaching; and 3) Health and lifestyle as peripheral coaching responsibilities. Table 1 illustrates how selected analytical codes were organised into subthemes and further developed into these final themes. Rather than serving as an exhaustive codebook, the table is intended to provide transparency regarding the analytical structure underpinning the findings (Table 1).

**Table 1:** Development of themes from selected codes and subthemes.

Illustrative empirical focus	Analytical codes	Subthemes	Themes
Coaches described being needed as stable adults and role models rather than as the best players Coaches described sofa talks, debriefs, and feedback after gameplay	Individualisation for the players Communication as a key component Feedback-oriented Creating safe spaces Coach as a role model	Adapting coaching through relational understanding Fostering safe and inclusive participation Modelling and supporting positive youth development	The grassroots esports coach as a youth-oriented facilitator
Coaches described limited equipment, few volunteers, and difficulties expanding activity Coaches described using YouTube, social media, websites, reviews of recorded gameplay, and peer discussion for learning	Lack of structure Scarce game-specific knowledge Lack of coaches and volunteers Equipment cost Need for paid or specialised coaching support	Balancing low-threshold participation and structured development Lack of formal esports competency pathways Fragile volunteer-based capacity Economic and material barriers to sustained activity	Resource capacity and structure in grassroots esports coaching
Coaches described sleep, nutrition, and physical activity as relevant but difficult to integrate Coaches described energy drinks as clearly prohibited during sessions	Physical activity, but unstructured Limited or inaccurate nutrition guidance Time constraints Players not responsive to advice Not part of the coaching role	Defining health as outside the core coaching role Health promotion as informal rather than systematic Practical and motivational constraints on lifestyle guidance	Health and lifestyle as peripheral coaching responsibilities

### 3.2 Theme 1: the grassroots esports coach as a youth-oriented facilitator

This theme describes how coaches perceived their role in grassroots esports as extending beyond game-specific instruction. A dominant pattern in the data was the coaches' lack of extensive competitive esports backgrounds, although familiarity with playing computer or console games was common. For most coaches, role legitimacy was therefore not primarily constructed through superior in-game ability, but through being a consistent and dependable adult presence and organising a safe and meaningful activity. A prominent perspective across the interviews was that coaches did not need to be better than the players in the specific esports title. Instead, they emphasised the importance of representing an adult presence, a role model, and an organiser of structured activity:

“I now have several players who are better than me at the game, so you don't necessarily have to be better than them. But they need an adult, a role model, someone who can be there and organise things. That is probably the most important part. And continuity — being stable, always present, and involved. That is what I feel the role is.” (Liam)

This account illustrates how coaching authority was often grounded in social and organisational responsibility rather than technical superiority. The coach was positioned as someone who made participation possible and dependable, rather than as the most skilled person in the room. Several coaches also connected the value of grassroots esports to its social dimension. Activities such as team-building exercises, physical activity, and trips to events were described as fundamental parts of the coach's facilitation role, helping to create belonging and social relationships among young players:

“There is a completely different sense of community when we all travel together, compared with only meeting once or twice a week for training. The social events add a lot. I can see new friendships forming, even among children who did not know each other before. That is also a key part of why we are here.” (Ethan)

Such accounts suggest that coaches understood grassroots esports as more than repeated game practice. Social events and shared experiences were framed as mechanisms for building group cohesion, reducing isolation, and transforming gaming into a collective club-based activity. Several coaches also emphasised communication as a key component of being a good role model and facilitator. A recurring strategy during training sessions was the use of informal debriefs, such as taking players or the whole team aside to summarise the session, provide feedback, and invite players to reflect on their own experiences:

“We have something that works really well: when you are finished with the game, we go over to the sofa and have a kind of sofa talk. It is a bit informal, just away from the game and away from all the different elements that can be involved. And then we get to give feedback to them, but also receive feedback from them.” (Henry)

The “sofa talk” example shows how some coaches created deliberate pauses between gameplay and reflection. These informal debriefs functioned as a practical pedagogical tool, allowing coaches to address behaviour, learning, and group dynamics without relying on formal coaching models. Because communication was viewed as central to esports training, coaches described toxic communication as incompatible with safe and inclusive participation and as something that had to be addressed immediately when it occurred. There was absolute agreement among all coaches that toxic behaviour should be strictly prohibited and addressed immediately when it occurred:

“Yes, I shut it down as well. I can tolerate a lot, because they are young people, but there are certain things I simply will not accept. For example, if someone starts shouting racist remarks, I make it clear: “I don't care who says it — I will not allow that here.” That kind of behaviour stops immediately.” (Alexander)

This was one of the clearest areas of consensus in the dataset. While coaches differed in their levels of game-specific expertise and session structure, they consistently framed toxic communication as a boundary issue that required immediate adult intervention. Finally, a key reflection among the participants was how their understanding of the coaching role had changed over time. Rather than applying a one-size-fits-all approach, they increasingly emphasised the need to adapt their coaching to individual players' needs, preferences, and developmental trajectories:

“When you first start out as a coach, it may be easy to think in absolutes, or in a rather black-and-white way. (..) You gradually come to understand that there are many different ways to reach the same goal. (..) What is white for one person may be black for another.” (Lisa)

This reflection illustrates a shift from rule-based or uniform coaching towards a more relational and adaptive understanding of the role. Across these accounts, grassroots esports coaching was described less as the delivery of a fixed training model and more as an ongoing process of adjusting structure, expectations, and communication to different players. However, while coaches embraced this complex role as social facilitators, translating these ideals into practice was often complicated by the realities of the grassroots environment. As the next theme demonstrates, these social ambitions frequently had to be navigated within a context characterised by limited resources and a lack of formal structure.

### 3.3 Theme 2: resource capacity and structure in grassroots esports coaching

This theme describes how grassroots esports coaching was shaped by resource capacity in a broad sense, including equipment, funding, adult involvement, coach competence, and access to practical training resources. While coaches valued the low-threshold and inclusive character of grassroots esports, they also described how limited capacity constrained what they were able to provide. The theme therefore captures how organisational resources and pedagogical structure were closely connected in everyday coaching practice. Across the dataset, limited capacity was not described as a single problem, but as a cluster of connected constraints that shaped who could participate, how often activities could be offered, and how structured the sessions could become. A recurring barrier was the financial cost of equipment when grassroots initiatives were growing:

“I wish I had known that the club would need a bit more money for equipment. Over the past month, we have had many new participants join. It would have been helpful to have more funds available in the club so that we could buy additional equipment. Fortunately, we received funding last week, which allowed us to buy two new monitors, new headsets, and two new controllers.” (Mike)

This example shows how growth itself could create new vulnerabilities for grassroots esports initiatives. Increased interest from players did not automatically translate into expanded activity unless clubs could also secure the necessary hardware, gaming peripherals such as keyboards, mice, and headsets, and funding. Limited access to equipment also affected players' opportunities to practise at home, which was seen as a hurdle for those who wanted to improve and develop their skills further:

“You do not need to have a PC at home, but of course, if you want to become good, you need to practise. If you do not have the opportunity to practise at home, and we only have one or two training sessions a week, then that is obviously quite limited. For some, buying a PC for 2,000 euros may be a big step.” (James)

This highlights a tension between low-threshold participation and skill development. While club sessions could provide access to organised activity, unequal access to equipment outside the club could still shape players' opportunities for progression. A pervasive challenge across the dataset was carrying substantial responsibility with limited support, often as one of only a few adults involved in the activity. Recruiting volunteers and adult coaches was consistently described as difficult, which limited both the quality and frequency of what the grassroots esports initiative could offer:

“Recruiting volunteers is difficult. Recruiting adult coaches is difficult. We are still essentially the core group that started it all. (..) We have discussed this in our meetings: if we want to offer more activities and be open on more days, we need more volunteers.” (James)

Here, volunteer availability emerged as a pedagogical constraint, not merely an organisational one. When few adults were involved, coaches had less capacity to provide individual follow-up, differentiated activities, or more frequent sessions. Coaches' accounts of training-session organisation suggested two broad approaches. The first approach utilised a loose structure, typically consisting of an overall timeframe, broad activity blocks, and planned routines for opening and closing the session:

“We always start with physical activity for up to 30 min. (..) Before we start on the computers, we have a briefing where we talk a little about what we are going to focus on. (..) Thirty minutes of physical activity, perhaps around 15 min of discussion in total, and then one hour of gaming.” (Elias)

In these accounts, structure did not necessarily mean highly formalised coaching. Rather, it often consisted of predictable routines, transitions, and broad activity blocks that helped give sessions a developmental and social frame. The second approach, however, lacked a defined strategy. Here, the sessions were loosely organised, and the adults appeared as facilitators navigating the sessions as they unfolded, rather than functioning as traditional esports coaches:

“No, I don't really have a strategy. It's more that I ask them about something, and then I help them. Or if they see that I'm struggling a bit with something, I try to help a little in relation to the question.” (Oliver)

This contrast suggests that “training” in grassroots esports could range from lightly structured pedagogical activity to free play with adult support. The difference reflected not simply whether coaches cared about player development, but whether they had the time, competence, resources, and practical tools to translate their developmental intentions into structured sessions. A central tension was that extensive game-specific competence was widely viewed as non-essential for providing meaningful training sessions, while it was simultaneously recognised that deeper game knowledge could improve the ability to support players' in-game development:

“But you can still make sure that the children experience good training sessions. You don't need game-specific competence to achieve that. However, if they are to develop their skills specifically within the game, (..) then there is a major difference between playing superficially after having learned about it [the game], and having thousands of hours of experience [in the game].” (Elias)

This tension was important because it separated two dimensions of the coaching role: the ability to facilitate a safe and meaningful group activity, and the ability to support specialised game-specific improvement. Several coaches indicated that the former could be achieved without elite-level game expertise, whereas the latter required deeper title-specific knowledge.

Most coaches described having little or no formal coach education. This may help explain why descriptions of session planning, player development, and feedback practices were often relatively broad, informal, and grounded in personal experience rather than formal coaching models. Where coaches had completed formal coach education, this was typically esports-specific, such as a game-specific coaching course or a basic introductory (i.e., game-agnostic) esports coaching course. Consequently, a strong need was articulated for more resources and guidance to support everyday coaching practice:

“It would also be useful to have a good way of introducing new coaches, for example by providing an example of how to structure a training session well. What does a good training session actually look like at its core? Having some concrete examples of that would be really useful.” (Theodore)

The request for concrete examples suggests that coaches were not only asking for abstract knowledge about coaching, but for practical resources that could make their role more manageable in everyday volunteer settings. Social media and websites functioned as the primary sources for learning about esports coaching strategies. However, a clear preference was expressed for discussion-based learning, either through conferences or coaching courses, where they could exchange experiences and discuss practical challenges with others:

“That is where you meet people from all over the country, at least those who are interested in knowledge sharing. There are so many skilled people there, and you can discuss specific situations with them. You spend the whole day together, so it has definitely been incredibly valuable for me in terms of gaining information.” (Theodore)

This preference for peer discussion indicates that coaches valued knowledge that was situated, practical, and responsive to local problems. Informal online resources could provide ideas, but coaches often described learning from other practitioners as more useful for interpreting and adapting knowledge to their own context.

This overarching lack of formal structure, combined with limited time and resources, inevitably forced coaches to prioritise certain aspects of their role over others. The key analytical point is not simply that grassroots esports lacked resources, but that limited material, volunteer, and pedagogical capacity shaped the boundaries of what coaches could realistically take responsibility for. Consequently, as the final theme illustrates, more holistic responsibilities—such as promoting physical health, nutrition, and a healthy lifestyle—were often pushed to the periphery of their coaching practice.

### 3.4 Theme 3: health and lifestyle as peripheral coaching responsibilities

This theme reflects how health and lifestyle were recognised as relevant, but not central, aspects of grassroots esports coaching. Coaches occasionally addressed topics such as sleep, physical activity, nutrition, and general wellbeing, but these were handled informally rather than as structured parts of training. Limited time, varying player receptiveness, and uncertainty about whether health promotion belonged within the coach role made lifestyle guidance a peripheral coaching responsibility. Because these topics were explicitly included in the interview guide, the analytical significance of this theme lies not in the mere presence of health-related talk, but in how coaches positioned such topics at the margins of their perceived mandate.

The prevailing approach to health-related aspects such as nutrition, sleep, and physical activity was characterised by a lack of structured focus. This was often attributed to limited time and to a perception that these topics might be more relevant for older players:

“At the moment, my team only meets for two hours per week, so there is limited time to cover those topics [physical activity, nutrition, and sleep]. There is only so much we can influence in that time, and some of them also take part in other activities outside the club. So yes, it is definitely something that should be addressed, but I think it becomes more relevant as they get a bit older.” (William)

This account illustrates how coaches could recognise the relevance of health-related issues while still placing them outside the practical centre of weekly coaching. Time constraints, player age, and limited influence beyond the club session were used to explain why such topics were difficult to integrate systematically. For some coaches, nutrition, physical activity, and sleep fell largely or entirely outside the esports coaching role. Instead, it was suggested that such work should be handled by personal trainers or other specialists:

“If I hear someone say, “I've only slept for two hours,” for example, I try to talk to them about it. But I don't really think it is my place to do so as a head coach. I think that is more the role of a personal trainer or lifestyle coach.” (Christian)

This illustrates a boundary-drawing process in which coaches distinguished between informal care or concern and specialised health or lifestyle guidance. The issue was therefore not only lack of knowledge, but uncertainty about mandate and role responsibility. An additional challenge highlighted was a lack of interest from the players. Even when coaches attempted to highlight health-promoting aspects, they found themselves having to adapt their coaching approach to accommodate the players' disinterest:

“They often do not care enough about it. I always try to promote a healthy lifestyle, including getting enough sleep, and I take that into account in my coaching. For example, if players have been playing matches after midnight, I am not going to make them do a review session afterwards.” (Callum)

This example shows how health-related considerations sometimes entered coaching indirectly, through adjustments to training load or expectations, rather than through explicit teaching or structured lifestyle education. Overall, nutrition, sleep, and physical activity appeared to occupy a marginal position within esports coaching practice. Apart from the inclusion of physical activity in some training sessions, these areas were rarely described as structured parts of their coaching. Although their importance was acknowledged, the data contained few examples of concrete or systematic guidance given to players:

“I try to motivate them around sleep, eating healthily, and choosing sugar-free drinks instead of sugary soft drinks. I try to encourage those choices.” (Liam)

Where such guidance occurred, it was typically framed as encouragement or informal advice rather than as part of a planned coaching curriculum. This reinforces the interpretation that health and lifestyle were recognised as relevant, but still weakly systematised within everyday practice. In the rare cases where nutrition was addressed, the advice was framed in practical and informal terms. Rather than reflecting structured or evidence-based nutrition guidance, such advice appeared to be based on coaches' own experiences and understandings of what might help players during competition:

“They are also encouraged to always have chocolate during matches, because it is a kind of “all-in-one solution” (..). The reason I mention chocolate is that it provides quick carbohydrates if you are feeling a bit tired and mentally slow, which can happen during a match.” (Lisa)

This example illustrates the informal and experience-based character of the limited nutrition guidance described in the interviews. It also shows why health-related guidance may require clearer boundaries and support if coaches are expected to address such topics. Despite the absence of systematic nutrition guidance, there was one absolute rule agreed upon by every coach: energy drinks were strictly prohibited during esports training sessions:

“Energy drinks are essentially not allowed.” (Noah)

Energy drinks therefore represented a distinct exception within the health theme. Unlike sleep, nutrition, or physical activity, energy drink use could be translated into a simple and enforceable rule within the club setting.

In summary, while coaches recognised the general relevance of health and lifestyle factors, the practical realities of grassroots esports dictated a different focus. With the notable exception of enforcing bans on energy drinks, health promotion remained a peripheral, informal, and largely unsystematised aspect of their role. The theme therefore shows how volunteer coaches drew boundaries between what they could regulate directly during sessions, what they could address through informal encouragement, and what they perceived as requiring expertise or support beyond the grassroots coaching role.

## 4 Discussion

This study explored how grassroots esports coaches understand and describe their roles in organised grassroots esports settings. Consistent with the interpretative design of the study, the findings should be understood as an analysis of coaches' role understandings rather than as an evaluation of whether their practices met a predefined model of effective coaching. The findings suggest that these coaches primarily understand their role as youth-oriented facilitators rather than as game-specific performance specialists. Across the interviews, coaches emphasised adult presence, social belonging, communication, safe boundaries, and individual adaptation as central to their practice. However, this facilitative role was understood within a context marked by limited formal structure, fragile volunteer capacity, equipment-related barriers, and a lack of established competency pathways. Health and lifestyle issues were recognised as relevant, but generally remained peripheral, informal, and weakly integrated into coaching practice. Taken together, the findings point to a grassroots coaching role defined by a tension between broad social responsibility and limited volunteer capacity. Rather than indicating a simple deficit in coaching practice, this tension illustrates how volunteer coaches adapt their role to the practical and organisational conditions of grassroots esports.

### 4.1 The social mandate of grassroots esports: redefining coaching expertise

A central finding of the present study is that grassroots esports coaches conceptualised their role primarily as youth-oriented social facilitation rather than game-specific technical instruction. This emphasis on safe participation, belonging, and role modelling aligns with recent research framing grassroots esports as a platform for social inclusion and youth development (7). It further resonates with positive youth development (PYD) literature, which stresses that developmental outcomes depend on intentional adult facilitation rather than mere participation (19–21). Recent work on scholastic esports coaching has similarly suggested that coaches may understand organised esports as a setting for cultivating life skills, social belonging, and positive peer environments (14). The present study extends this discussion to volunteer grassroots esports by showing how similar developmental ambitions are articulated in less formalised and more resource-constrained club and community settings.

However, the coaches' developmental vocabulary was notably selective. Whereas youth sport coaching literature often describes PYD responsibilities in broad terms, including life skills, confidence, and character development (19, 20), the coaches in the present study tended to describe their developmental role in more immediate and practical terms. They primarily emphasised foundational youth-work: connection, safe climates, and the mitigation of toxic behaviour. This should not necessarily be interpreted as a lack of developmental awareness among the coaches. A more reasonable interpretation is that, in voluntary grassroots contexts, coaches prioritised the most immediate conditions for participation: ensuring that young people had a safe place to meet, communicate, and play together. In this sense, social facilitation may represent a contextually appropriate adaptation to grassroots esports rather than a weaker form of coaching. The relative absence of explicit language around psychological developmen tmay reflect an emerging, weakly formalised coaching field where volunteers must prioritise immediate psychosocial safety over long-term life-skills transfer.

Côté and Gilbert's (17) framework of coaching effectiveness is useful for interpreting how coaches described the knowledge base of their role. The coaches did not present game-specific expertise as the only foundation for coaching. Instead, they emphasised social, organisational, and pedagogical dimensions, such as being present, creating structure, communicating with players, and supporting a safe environment. At the same time, the findings do not suggest that game-specific knowledge was irrelevant. Rather, coaches described a tension between the idea that meaningful grassroots sessions can be facilitated without being the most skilled player and the recognition that deeper game knowledge becomes more important when the aim is specialised skill development. This tension aligns with esports coaching research suggesting that gameplay experience remains an important marker of credibility (12), while traditional coaching literature cautions that playing expertise does not automatically translate into coaching ability (24, 38, 39). The finding extends esports coaching research conducted largely in performance-oriented settings, where both game-specific expertise and relational dimensions of coaching have been identified as important. In the volunteer grassroots contexts examined here, however, coaches placed particular emphasis on continuity, relational trust, and safe participation alongside game-specific knowledge. Their accounts therefore suggest that coaching credibility was understood not only in terms of what a coach knew about the game, but also in terms of their capacity to create stable, inclusive, and responsive environments. This emphasis on adapting communication, expectations, and session structure to individual players resonates with athlete-centred coaching perspectives, where effective coaching is understood as relational, responsive, and attentive to participants' needs and development rather than solely as the delivery of technical instruction.

Ultimately, our findings point to a potential role ambiguity. Esports coaching appears as a distinct practice shaped by unique digital and cultural conditions, not merely a digital translation of traditional sport coaching (10). While coaches can foster meaningful social participation without being superior players, game-specific knowledge may become increasingly important when the objective shifts toward specialised skill acquisition (11). The present study therefore illuminates how grassroots esports volunteers understand and describe their roles in an ambiguous coaching landscape, while leaving open whether players themselves value social facilitation over technical expertise. This distinction is important for coach education: grassroots coaches may need support that recognises both dimensions of the role, rather than programmes that assume either that game expertise is sufficient or that generic youth facilitation alone can address all coaching demands in esports.

### 4.2 The capacity challenge of grassroots esports coaching: material, volunteer, and pedagogical resources

The findings reveal a capacity challenge in voluntary grassroots esports: coaches are expected to facilitate inclusive and meaningful activities while operating with limited material, volunteer, and pedagogical resources. This capacity challenge is central to interpreting the findings because it shaped not only what clubs could offer materially, but also what kinds of coaching practices are realistic.

Financial constraints should be understood as more than general organisational limitations. In the voluntary and club-based settings examined here, resources shaped whether clubs could provide the material conditions for participation and practice. Whereas youth sport participation is often affected by fees, travel, competition, and equipment costs (40, 41), grassroots esports depends on a distinct digital infrastructure: high-end devices, peripherals, and network stability. The present study extends community sport capacity research by showing how digital participation remains materially dependent and may reproduce inequalities in access and development when clubs or players lack adequate equipment (42, 43). Material constraints were also compounded by limited volunteer capacity. In line with recent research on grassroots esports clubs, where volunteer recruitment, financial limitations, and insufficient adult involvement restrict sustainable activity (7), the present study shows that limited availability of volunteers constrained pedagogical practice by reducing session frequency, individual follow-up, and systematic player support (44). The contribution here is to show that material, volunteer, and pedagogical capacity were not separate issues. According to coaches' accounts, they interacted: limited equipment constrained access, limited volunteer availability constrained follow-up, and limited pedagogical resources constrained the ability to turn gaming sessions into more intentional training environments.

Beyond material and organisational constraints, coaches also faced a competency gap. Professional esports coaching has been described as weakly formalised, with uncodified education pathways, limited best-practice guidance, unclear standards, and few established methods for evaluating coaching effectiveness (9, 10). The present study extends this discussion to the grassroots level by showing that volunteer coaches lacked not only formal esports-specific education, but also basic pedagogical tools such as session templates, onboarding resources, and models for balancing social facilitation with game-specific development. Coaches described relying on informal and self-directed learning sources, including YouTube, social media, websites, search engines, peer discussion, and reviews of recorded gameplay, to support game-specific learning. Rather than being anchored in a clear esports-specific education pathway, coaches' learning appeared to be distributed across formal, non-formal, and informal sources (45). Peer-learning arenas align with communities of practice perspectives, where learning is understood as situated, social, and developed through participation in shared practice (46, 47). However, reliance on open online platforms also requires source-critical competence, particularly when guidance extends beyond game mechanics (48, 49). This finding illustrates a productive tension. Informal and peer-based learning may be well suited to a rapidly changing digital field where game knowledge evolves quickly, but it may also leave volunteer coaches without shared standards, pedagogical language, or reliable guidance on topics that extend beyond gameplay.

The pedagogical consequence of this capacity challenge was visible in the variation between coach-structured esports training and free play. Free play may support social belonging and low-threshold participation, but skill development requires more than repeated gameplay; purposeful practice depends on clear intentions, feedback, repetition, and coach oversight (11, 13, 23, 50–52). At the same time, free play should not be dismissed as pedagogically meaningless in grassroots contexts. For some participants, low-threshold access, adult presence, and social belonging may be central outcomes in themselves. The challenge is therefore not to replace grassroots participation with elite-performance logics, but to help volunteers distinguish between when free play is sufficient for social participation and when more deliberate session structure is needed to support learning and development. Grassroots esports development therefore requires both material support and accessible coach-development resources that help volunteers plan, structure, and reflect on their practice. The goal should not be to impose elite-performance logics on voluntary clubs, but to make the coaching role more accessible, sustainable, and pedagogically coherent.

### 4.3 The peripheral health mandate of grassroots esports coaching: lifestyle guidance at the edge of coaching expertise

Because coaches were explicitly asked about health-related topics, the significance of this theme does not lie in the mere presence of sleep, nutrition, physical activity, and energy drink use in the interviews. Rather, the key finding is how coaches positioned these issues at the margins of their role. While coaches recognised that health and lifestyle could be relevant to esports participation, they generally framed such guidance as difficult to integrate, secondary to their core responsibilities, or partly outside their expertise. Volunteer grassroots coaches may occupy roles that partly overlap with health and lifestyle support, while still lacking the training, confidence, and mandate to provide specialised guidance. The analytical question is therefore not whether they provided comprehensive health guidance, but how they drew boundaries between manageable activity rules, informal support, and issues that require clearer education, resources, or referral pathways. This framing is important because it avoids treating health promotion as an assumed duty of volunteer coaches. Instead, the findings show how coaches themselves negotiated the boundary between care, common-sense advice, and specialised health guidance.

The perceived low relevance of such topics among players sits uneasily with esports health literature documenting pain and overuse complaints, suboptimal dietary patterns, insufficient physical activity, and poor adherence to general health recommendations among players (25, 26, 53, 54). Previous research on Norwegian esports students similarly indicates that players may recognise the performance relevance of sleep and nutrition while still reporting suboptimal habits (28). The marginalisation of health guidance should therefore be understood as a structural capacity and role-definition problem, rather than evidence that lifestyle factors are unimportant in grassroots esports. However, the findings also suggest that making health and lifestyle guidance more visible in grassroots esports requires careful boundary-setting. If such topics are introduced without clear resources, referral pathways, or role clarification, this may risk expanding volunteer coaches' responsibilities beyond what is realistic or appropriate.

Energy drinks represent a notable exception to this peripheral health mandate. The issue is contextually relevant in Norway: recent public health data show that 46% of adolescents report weekly consumption of caffeinated energy drinks, while earlier Norwegian adolescent data link energy drink consumption to high leisure-time screen use (55, 56). Paediatric and public health literature also raises concerns about such consumption among children and adolescents (57, 58), and regular use has been reported among Norwegian esports students (28). Analytically, the coaches' consistent ban on energy drinks is revealing. Unlike sleep or physical activity, which require complex and sustained lifestyle guidance, energy drinks are tangible products that can be regulated through simple, enforceable boundaries during sessions. The energy drink example suggests that grassroots esports volunteers can understand health-related responsibilities when these can be translated into manageable structural rules rather than broad behavioural interventions. This distinction helps explain why energy drinks were treated differently from sleep, nutrition, or physical activity. The issue of energy drinks could be managed as a session rule within the coach's authority, rather than as an attempt to influence players' broader lifestyle outside the club.

When coaches occasionally provided lifestyle guidance, such as advising players to eat chocolate for quick energy, their advice was largely unsystematic and grounded in personal anecdote rather than evidence-informed knowledge. Sports nutrition research demonstrates that athletes often rely on coaches for dietary information, while coaches' nutrition knowledge is frequently variable and lower than that of trained health or performance-support personnel (59, 60). In grassroots esports, where coach education relies heavily on informal online ecologies, this knowledge gap represents a significant challenge. Effective guidance requires eHealth literacy: the ability to find, appraise, and apply digital health information appropriately (48). Given that online health information is often misleading or false (49), reliance on informal sources places lifestyle guidance at the limits of grassroots coaches' expertise. Rather than implying that volunteer coaches should become health professionals, this finding suggests a need for bounded and evidence-informed support: practical guidance on what coaches can reasonably address, when they should refrain from providing specialised advice, and when referral to parents, clubs, or qualified professionals may be more appropriate.

### 4.4 Practical implications

The findings suggest that federations, clubs, and policymakers should prioritise accessible and practical support structures for coaches rather than extensive professionalisation. Given the exploratory and interpretative nature of the study, these implications should be read as practice-oriented suggestions rather than prescriptive recommendations. Practical support for volunteer coaches could include introductory coach education that clarifies the grassroots coaching role, practical session templates, and onboarding materials for new volunteers. These resources may help coaches structure activities without requiring extensive prior experience or elite-level game expertise.

Because coaches relied heavily on informal online resources and peer discussion, federations and clubs should also facilitate peer-learning arenas where volunteers can share experiences, discuss practical challenges, and adapt coaching knowledge to local contexts. Practical guidance should address communication, toxic behaviour, online conduct, inclusion, and safeguarding, as these were central to how coaches understood safe and meaningful participation.

Finally, health and lifestyle guidance should be clearly bounded. Coaches should not be positioned as health professionals, but they may benefit from evidence-informed resources, skills for evaluating online sources, basic eHealth literacy, and clear referral pathways on topics such as sleep, breaks, physical activity, nutrition, and energy drinks. Optional game- or genre-specific modules could then be added for clubs seeking more specialised skill development. More broadly, these findings have implications for sport policy, digital youth engagement, and coach education systems, as grassroots esports coaching appears to require support structures that recognise its hybrid position between sport coaching, youth work, digital culture, and community volunteering.

## 5 Strengths and limitations

A distinct strength of this study is its provision of in-depth qualitative insight into a largely under-researched group: volunteer grassroots esports coaches. By including 17 coaches from different clubs across Norway, Denmark, and the Netherlands, the analysis captures variation across organisational contexts while maintaining a focus on shared challenges in voluntary, club-based esports. The study also contributes to the field by connecting coaching role perceptions with organisational capacity, competency development, and health-related responsibilities, areas that remain weakly developed in existing esports coaching literature. Methodologically, the study provides detailed reporting of recruitment, participant context, data collection, transcription, and analytic procedures, supporting transparency and enabling readers to assess the transferability of the findings. The use of participant quotations and an analytical table further helps make visible how the interpretation was grounded in the empirical material. A further strength is that the interviewers had several years of esports experience, which supported context-sensitive follow-up questions and interpretation of esports-specific terminology. At the same time, this insider familiarity required reflexive awareness of how the researchers' prior experiences and professional backgrounds may have shaped data collection, analysis, and interpretation.

Several limitations must also be acknowledged. First, the sample was unevenly distributed geographically, with Norwegian coaches overrepresented compared with Danish and Dutch participants. Because the study was not designed as a comparative cross-national analysis, and because the country subsamples were small and uneven, the findings should not be interpreted as evidence of national differences in grassroots esports coaching. Although country was considered as contextual information during analysis, no consistent country-specific patterns were identified. Because participation was voluntary and based on convenience sampling, the sample may also overrepresent coaches who were particularly engaged in esports development or motivated to discuss coaching practice. Second, the sample was strongly gender-imbalanced, with only one woman among the 17 participants. The findings may therefore insufficiently capture gendered experiences of esports coaching, and the recruitment process may have been affected by gender-based selection bias within a heavily male-dominated field. Finally, years of coaching experience were not used as an inclusion criterion and were not systematically collected. This limits the study's ability to examine how coaching experience may have shaped participants' role understanding, pedagogical practices, and perceived competence needs.

Future research should incorporate players' perspectives, particularly to examine whether players value social facilitation over game-specific expertise and whether their views align with coaches' perception that players are unreceptive to lifestyle guidance. Observational studies could further examine how coaching roles, session structures, and health-related boundaries are enacted in practice, while mixed-methods or survey designs could assess how widespread these patterns are across clubs, countries, genders, game titles, and organisational models. Future comparative research should examine how national sport systems, esports ecosystems, voluntary-sector traditions, and governance structures shape grassroots esports coaching. Other studies could examine player and coach motivation in grassroots esports more directly, for example through motivational frameworks such as Self-Determination Theory (61). Finally, as is inherent to reflexive thematic analysis, the themes presented here represent one theoretically informed interpretation of the data rather than a definitive account of all possible patterns.

## 6 Conclusion

This study provides qualitative insight into how volunteer grassroots esports coaches understand and negotiate their roles in organised, club-based esports. Across Norway, Denmark, and the Netherlands, coaches positioned their work primarily as youth-oriented social facilitation rather than game-specific technical instruction. Their accounts emphasised adult presence, boundary-setting, communication, role modelling, and the creation of safe and inclusive environments. At the same time, the findings show that this social mandate is carried out under substantial material, organisational, and competency-related constraints. Limited equipment, volunteer capacity, and formal coach education shaped what coaches were able to provide and contributed to variation between structured esports training and more free play sessions.

The study also showed that health and lifestyle guidance occupied a marginal and weakly systematised position in grassroots esports coaching. Although coaches aimed to create developmentally meaningful environments, advice on nutrition, sleep, physical activity, and broader lifestyle issues was often perceived as outside the coaching role, inconsistently addressed, or based on personal experience rather than evidence-informed knowledge. Banning energy drinks represented a notable exception, suggesting that coaches found health-related responsibilities easier to address when they could be translated into clear and enforceable boundaries.

Overall, grassroots esports coaching appears to sit at the intersection of youth work, informal sport coaching, digital culture, and community volunteering. Given the exploratory and non-comparative design, these findings should be understood as analytically transferable rather than nationally representative. Strengthening this field does not require transforming volunteers into elite esports specialists or health professionals. Rather, grassroots esports needs material support, accessible coach education, peer-learning arenas, source-critical competence, and accessible practical resources that help volunteers deliver safe, inclusive, and pedagogically coherent activities. By positioning grassroots esports coaching within broader issues of sport policy, digital youth engagement, and coach education systems, the study highlights the need for support structures that recognise both the opportunities and constraints of voluntary esports provision.

## Data Availability

The datasets generated and analyzed during the current study are not publicly available due to confidentiality considerations and the potential risk of participant identification within a small and interconnected field. Selected anonymized excerpts are included in the manuscript. Further inquiries may be directed to the corresponding author, subject to applicable ethical and data protection restrictions. Requests to access the datasets should be directed to André Baumann at ab@gamersperformance.no.
