# Supplementary material for: Coaching beyond the game: a qualitative study of volunteer coaches in European grassroots esports
Source: Front Sports Act Living. 2026 Aug 28;8:1913015. doi: 10.3389/fspor.2026.1913015 (PMC13562047; doi:10.3389/fspor.2026.1913015)
Supplement: Supplementary file 1 [file Datasheet1.pdf]

Interview Guide

Suggested overarching research questions:

1. Coach identity and competence: How do grassroots coaches construct identity and authority in the absence of formal qualification requirements, and which sources of competence do they base their practice on?
2. Pedagogical practice in grassroots esports: How do coaches plan and conduct training sessions, and how do they balance performance, mastery, inclusion, and safety?
3. Adaptation from elite to grassroots esports: How are methods from elite esports, such as "review–plan–do", adapted to a grassroots context with social goals and the management of digital misconduct or toxicity?
4. Structural conditions and support: How do organisation and support structures influence the coaching role, and how is the "dual role" of being both a game coach and a youth leader experienced?
5. Needs and development: What do coaches need in order to succeed and to develop more sustainable, safe, and inclusive grassroots esports activities?

Semi-structured interview guide

Coach identity and competence: background, role, and motivation

Coach identity, competence base, and entry into the role. Why are they esports coaches? What background and reference points do they have in relation to gaming and esports, and possibly coaching more generally? What training have they received?

Can you tell me the story of how you became an esports coach?

• What was your pathway into a coaching role?

• What was your motivation? For example, your own interest in gaming, children in the club, or a spirit of volunteering?

• How do you use your own background and gaming experience in the coaching role?

• What experience do you have as a player, and at what level have you played?

• How do you use your own player experience when teaching others?

• Follow-up, based on Watson et al., 2025: To what extent do you think a person needs to have been good at the game themselves in order to be a good coach? Why or why not?

• What formal or informal training have you received in your role as a coach?

• Which coaching courses have you taken, for example through DGI, a sport federation, or the Norwegian coach education pathway?

• If none: Where do you go to find knowledge? For example, YouTube, Discord, or other coaches?

• How confident do you feel in the role as an adult leader? What do you think about the role of “game expert” versus “youth worker” or “social worker”?

• If you get stuck or need new knowledge about coaching or training, where do you turn?

• Can you describe which resources or networks you use?

• How do you assess the need for formal competence, such as courses or education, compared with “learning by doing” in your everyday practice?

Pedagogical practice

The balance between performance and grassroots/social values. How are training sessions conducted? Which courses have the coaches completed? How do they facilitate training in practice in relation to pedagogy and didactics?

Can you describe what a typical training session looks like?

• How do you start? How do you finish?

• How much time is spent on playing compared with discussion, analysis, or social activities?

• Which tools, such as reviews or watching recordings, do you use, and how much do you use these?

• How do you work pedagogically to develop the players’ skills?

• How do you handle differences in skill level within the group? For example, those who want to become professionals compared with those who just want to have fun. • How do you work to include those who may fall outside socially?

• Esports can sometimes have a harsh tone online, often referred to as toxicity. Have you experienced this in your group?

• How do you handle it if a player behaves badly or becomes angry, for example “tilts”? • Which “house rules” or ethical guidelines do you have or follow?

How do you work with topics beyond the game itself, such as physical health, sleep, and nutrition?

• To what extent do you talk with the players about these topics? • To what extent do you include physical activity as part of the training programme? Why or why not?

Inclusion, diversity, and the “social mandate”

Toxicity and environment: the broader social mission. How do coaches perceive the gender balance in the club? Why is the gender balance as it is? How do coaches explain participation in the club?

• How would you describe the social environment in your team?

• What do you do specifically to bring the group together and prevent social exclusion? • Many esports players may have social challenges or diagnoses, such as ADHD or autism. Is this relevant for the players you coach, and how do you adapt to this diversity in practice?

• What does the gender balance look like in your club?

• Do you have girls on the team?

• If no: Why do you think that is? Have you taken active measures to recruit girls?

• If yes: What have you done to help them feel comfortable and remain in a male-dominated environment?

• What do you think may explain the situation in your club?

Many people experience that esports attracts children and young people who may have fallen outside traditional sport, or who may have challenges, for example within the autism spectrum or ADHD.

• Is this something you recognise?

• How do you adapt the training sessions to accommodate this diversity? • What type of pedagogy does this require from you, for example compared with football or handball?

• Grassroots sport should be for everyone, regardless of financial background. Is equipment pressure, such as expectations around having a PC or equipment at home, a barrier to participation in your setting?

• To what extent can everyone participate regardless of background? What, if anything, is required from parents? • To what extent does the club have equipment available for borrowing?

Structural conditions and support

National models and organisational support. Is there a lack of support, both financial and otherwise? Where do coaches receive support from? What kind of support does the coach need?

• How do you experience the support from the club or organisation you are part of?

• To what extent does the board or leadership understand what esports is? • To what extent do you have the equipment and facilities you need? • For Norway/sports clubs: To what extent do you experience esports as a “fully integrated” part of the sports club, or as an activity on the side?

• How is the collaboration with the players’ parents?

• Do they understand what their children are doing? • To what extent do you experience scepticism, for example concerns about screen time, compared with support? • How do you communicate the value of esports to sceptical parents? • What attitudes do you encounter among parents regarding esports? • How do you explain the value of what you are doing to sceptical parents?

• Can you describe some of the challenges you encounter in everyday life as an esports coach?

• Time pressure? Technical problems? Lack of competence? Finances?

• Are there resources or tools that would make your job easier?

• For example, ready-made training sessions, a course in pedagogy, better PCs, or an assistant?

Closing and future development

• Are there any topics or challenges we have not covered that you think are important for understanding your role?

Demographic information

• How old are you?

• Do you identify as male or female?
